# Effect of preparation method for radioactive iodine therapy on serum electrolytes

**DOI:** 10.1007/s11604-023-01444-9

**Published:** 2023-05-15

**Authors:** Noriko Takata, Masao Miyagawa, Tomohisa Okada, Naoto Kawaguchi, Yutaka Fujimoto, Yoshihiro Kouchi, Shintaro Tsuruoka, Kotaro Uwatsu, Teruhito Kido

**Affiliations:** Department of Radiology, Ehime University Hospital, Shitsukawa, Toon, Ehime Japan

**Keywords:** Radioactive iodine therapy, Thyroid cancer, Hyponatremia, Hyperkalemia, Electrolytes

## Abstract

**Purpose:**

Thyroid hormone withdrawal (THW) in preparation for radioactive iodine therapy (RIT) may lead to hyponatremia and hyperkalemia because hypothyroidism reduces the glomerular filtration rate. Using recombinant human thyrotropin (rhTSH) may avoid these changes; however, these two preparation methods have not been compared in the literature. The purpose of this study was to reveal whether THW and rhTSH as preparation methods for RIT affect serum electrolytes differently. We also evaluated clinical factors influencing the onset of hyponatremia and hyperkalemia during RIT.

**Materials and methods:**

From April 2005 to December 2020, we analyzed 278 patients with thyroid cancer who received RIT. The patients were classified into two groups based on the preparation method, and renal function and serum electrolytes were compared between the groups. We also evaluated clinical factors that may affect overt hyponatremia (serum sodium level < 134 mmol/L) and hyperkalemia (serum potassium level ≥ 5.0 mmol/L).

**Results:**

Serum sodium and chloride levels in the THW group were significantly lower than those in the rhTSH group (p < 0.001 and p = 0.002, respectively). In contrast, the serum potassium level in the THW group was significantly higher than that in the rhTSH group (p = 0.008). As for clinical factors that may influence hyponatremia, age and estimated glomerular filtration rate (eGFR) were significantly associated with serum sodium level in the univariate analysis (p = 0.033 and p = 0.006, respectively). In the multivariate analysis, only age was significantly associated with serum sodium level (p = 0.030). Regarding hyperkalemia, distant metastases, the preparation method and eGFR were significantly associated with the serum potassium level in the univariate analysis (p = 0.005, p = 0.005 and p = 0.001, respectively). In the multivariate analysis, only eGFR was significantly associated with hyperkalemia (p = 0.019).

**Conclusion:**

THW and rhTSH affect serum sodium and potassium levels differently. Renal function may be risk factors for hyperkalemia, whereas older age may be a risk factor for hyponatremia.

## Introduction

Radioactive iodine therapy (RIT) is one of the treatments for patients with thyroid cancer; it is used as adjuvant therapy in patients with a high recurrence risk or as treatment for residual lesions or metastatic disease [1]. To achieve RIT successfully, iodine restriction and thyroid stimulating hormone (TSH) elevation are needed in preparation. Conventionally, TSH elevation has been achieved by thyroid hormone withdrawal (THW). However, this method causes temporary hypothyroidism because RIT is performed after total thyroidectomy. Hypothyroidism causes various symptoms, including a reduction in the glomerular filtration rate (GFR) [2]. Previous reports have shown that decreased renal function caused by THW for RIT may lead to hyponatremia and hyperkalemia [3, 4]. Moreover, patients are often instructed to drink more water during RIT to accelerate radioiodine excretion, which may also lead to hyponatremia [3]. Hyponatremia is defined as a serum sodium level < 135 mmol/L, and it is a common electrolyte disorder [5–7]. Acute severe hyponatremia (< 120 mmol/L) is often symptomatic and poses a high risk of neurologic complications such as cerebral edema [6, 7]. Although the frequency of severe hyponatremia during RIT is rare, several cases of life-threatening hyponatremia during the THW period for RIT have been reported [3]. On the other hand, hyperkalemia is defined as a serum potassium level > 5.0 mmol/L [8]. Severe hyperkalemia may cause fetal arrhythmia, and electrocardiographic (ECG) monitoring and acute intervention are recommended in patients with serum potassium levels > 6.5 mmol/L [8]. However, overt hyperkalemia during RIT is reportedly rare [4]. Nevertheless, the outcomes of severe hyperkalemia are serious, and this adverse event cannot be ignored.

Recombinant human thyrotropin (rhTSH), sold under the brand name Thyrogen®, was approved as an alternative method to THW in the 2000s [9]. Using rhTSH can decrease the problems associated with hypothyroidism [10]. Thus, changes in serum electrolytes, such as hyponatremia and hyperkalemia, may not be observed during RIT with the rhTSH method. However, a comparison between the rhTSH and THW methods has not been reported. Therefore, the purpose of this study was to reveal whether rhTSH and THW in preparation for RIT affect the serum electrolytes differently. We also evaluated clinical factors influencing the onset of hyponatremia and hyperkalemia during RIT.

## Materials and methods

### Patients

This retrospective study was approved by our institutional review board (No. 2207006), and we obtained informed consent from patients through an opt-out form on the website. From April 2005 to December 2020, 296 consecutive patients who received RIT at our institution were included in this study. The patients were classified into two groups according to preparation method.

All patients who received RIT until 2012 used the THW method. Since 2013, we have selected the preparation method based on the status of thyroid cancer, and the rhTSH method was used for patients without distant metastases. The THW method was started by changing levothyroxine (LT4) to liothyronine (LT3) 4 weeks before RIT, followed by discontinuing LT3 2 weeks before RIT. LT4 was restarted 4 days after RIT. Patients in the rhTSH group received 0.9 mg rhTSH injections 1 and 2 days before RIT. Blood samples were assessed within 7 days before RIT, including serum electrolytes, creatinine, estimated GFR (eGFR), thyroid hormone, and TSH. In the rhTSH group, blood samples were obtained on the day of RIT so that we can assess TSH elevation accurately after two injections of rhTSH. We calculated the eGFR based on revised equations for eGFR from serum creatinine in Japan [11]. Patients without appropriate laboratory data for analyses were excluded (n = 17). In addition, we excluded patients with primary aldosteronism, which may affect serum electrolytes (n = 1). Finally, we analyzed 278 patients with thyroid cancer. We compared renal function and serum electrolytes between the two groups.

### Statistical analysis

We used Student’s *t* test to compare blood sample data between the two groups, and a p-value < 0.05 was considered statistically significant. When we analyzed clinical factors influencing serum electrolytes, Student’s *t* test and Pearson’s chi-square test were used in the univariate analyses, and logistic regression analyses were used in the multivariate analyses. In our study, hyponatremia was defined as a serum sodium level < 134 mmol/L [12], and hyperkalemia was defined as a serum potassium level ≥ 5.0 mmol/L [8]. Statistical analyses were performed with JMP software (version 12.0; SAS Institute Inc., Cary, NC, USA).

## Results

In this study, 112 patients were categorized into the rhTSH group, and the remaining 166 patients were categorized into the THW group. Table 1 shows the baseline characteristics of the two groups. Patients in the THW group tended to be older (p = 0.001) with lower renal functions (p < 0.001) than those in the rhTSH group. The THW group included both patients with distant metastases (n = 117) and patients without distant metastases (n = 49). We compared whether there were differences in clinical factors between these two sub-groups. However, these factors were not significantly different except for age (p < 0.001).

**Table 1:** Relationship between the clinical characteristics of the two preparation groups

Parameters	Total (N = 278)	rhTSH group (N = 112)	THW group (N = 166)	P-value
Age (years)	62.4 ± 14.3 (20–87)	58.9 ± 15.4 (20–85)	64.8 ± 12.9 (22–87)	**0.001**
< 65	131 (47.1%)	63 (56.3%)	68 (41.0%)	**0.012**
≥ 65	147 (52.9%)	49 (43.8%)	98 (59.0%)	
Sex				
Female/male	173/105 (62.2%)	68/44 (60.7%)	105/61 (63.3%)	0.669
BMI	23.7 ± 3.9 (14.7–38.1)	23.5 ± 4.1 (14.7–38.1)	23.8 ± 3.7 (14.9–37.5)	0.548
≥ 25	84 (30.2%)	30 (26.8%)	54 (32.5%)	0.306
Distant metastases				
Yes	117 (42.1%)	0 (0.0%)	117 (70.5%)	** < 0.001**
Using ACEIs or ARBs				
Yes	66 (23.7%)	27 (24.1%)	39 (23.5%)	0.928
eGFR (mL/min/1.73m^2^)	62.8 ± 19.9 (9.6–167)	72.0 ± 20.8 (24.9–167)	56.5 ± 16.5 (9.6–120)	** < 0.001**
≤ 45	44 (15.9%)	10 (8.9%)	34 (20.5%)	**0.010**
Serum sodium (mmol/L)	140.0 ± 2.2 (130–145)	140.6 ± 2.2 (130–145)	139.6 ± 2.2 (132–145)	** < 0.001**
< 134	5 (1.8%)	2 (1.8%)	3 (1.8%)	0.989
Serum potassium (mmol/L)	4.3 ± 0.5 (2.9–6.1)	4.2 ± 0.4 (2.9–5.3)	4.3 ± 0.5 (3.2–6.1)	**0.008**
≥ 5.0	23 (8.3%)	3 (2.4%)	20 (12.0%)	**0.005**

Data for continuous variables are presented as mean value ± standard deviation (range)

*rhTSH* recombinant human thyrotropin, *THW* thyroid hormone withdrawal, *BMI* body mass index, *ACEIs* angiotensin-converting enzyme inhibitors, *ARBs* angiotensin II receptor blockers, *eGFR* estimated glomerular filtration rate

A p-value <0.05 was considered statistically significant, which is shown in bold

**Fig. 1:**
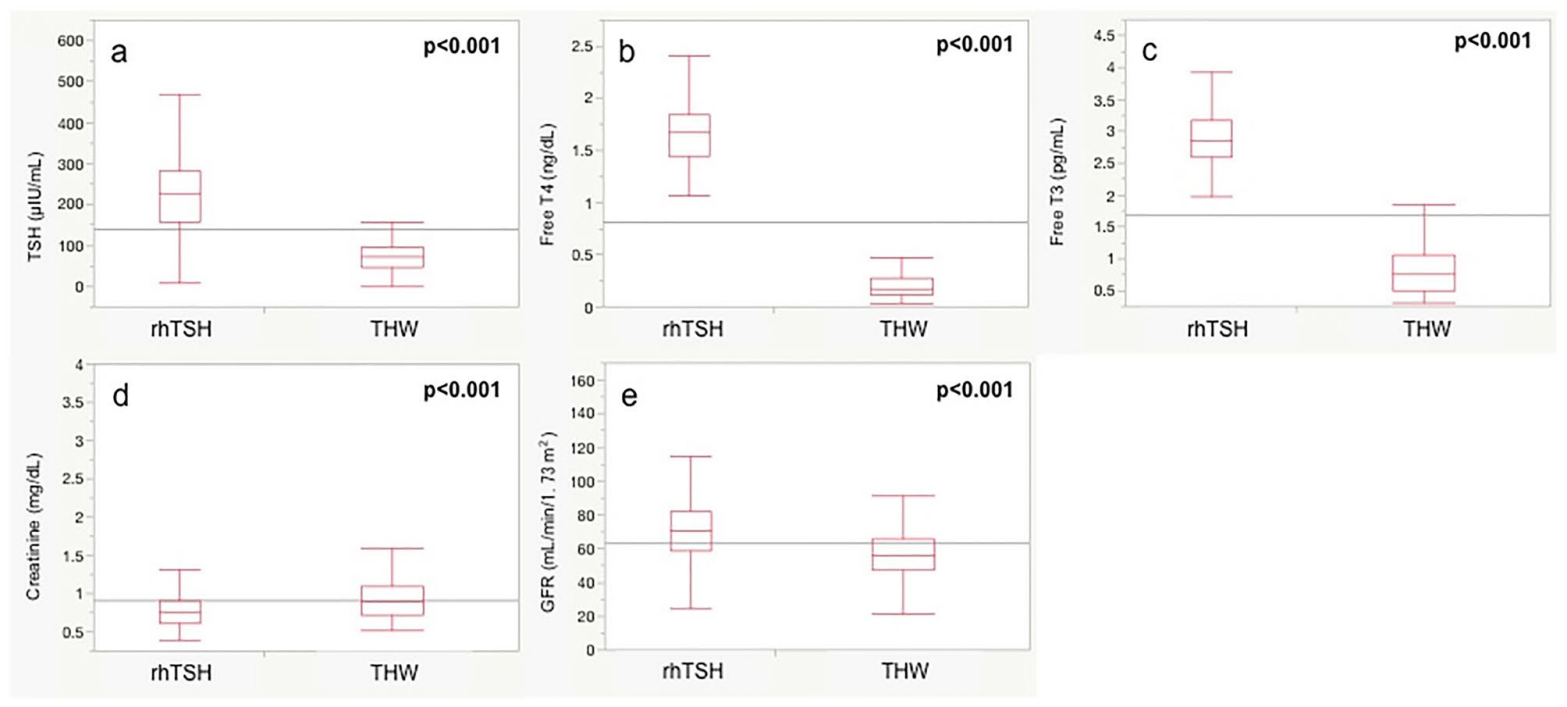
Differences in thyroid hormone and renal function between the two preparation methods for radioactive iodine therapy. **a** Thyroid stimulating hormone (TSH), **b** free T4, **c** free T3, **d** serum creatinine, and **e** estimated glomerular filtration rate (eGFR). *rhTSH* recombinant human thyrotropin, *THW* thyroid hormone withdrawal

**Fig. 2:**
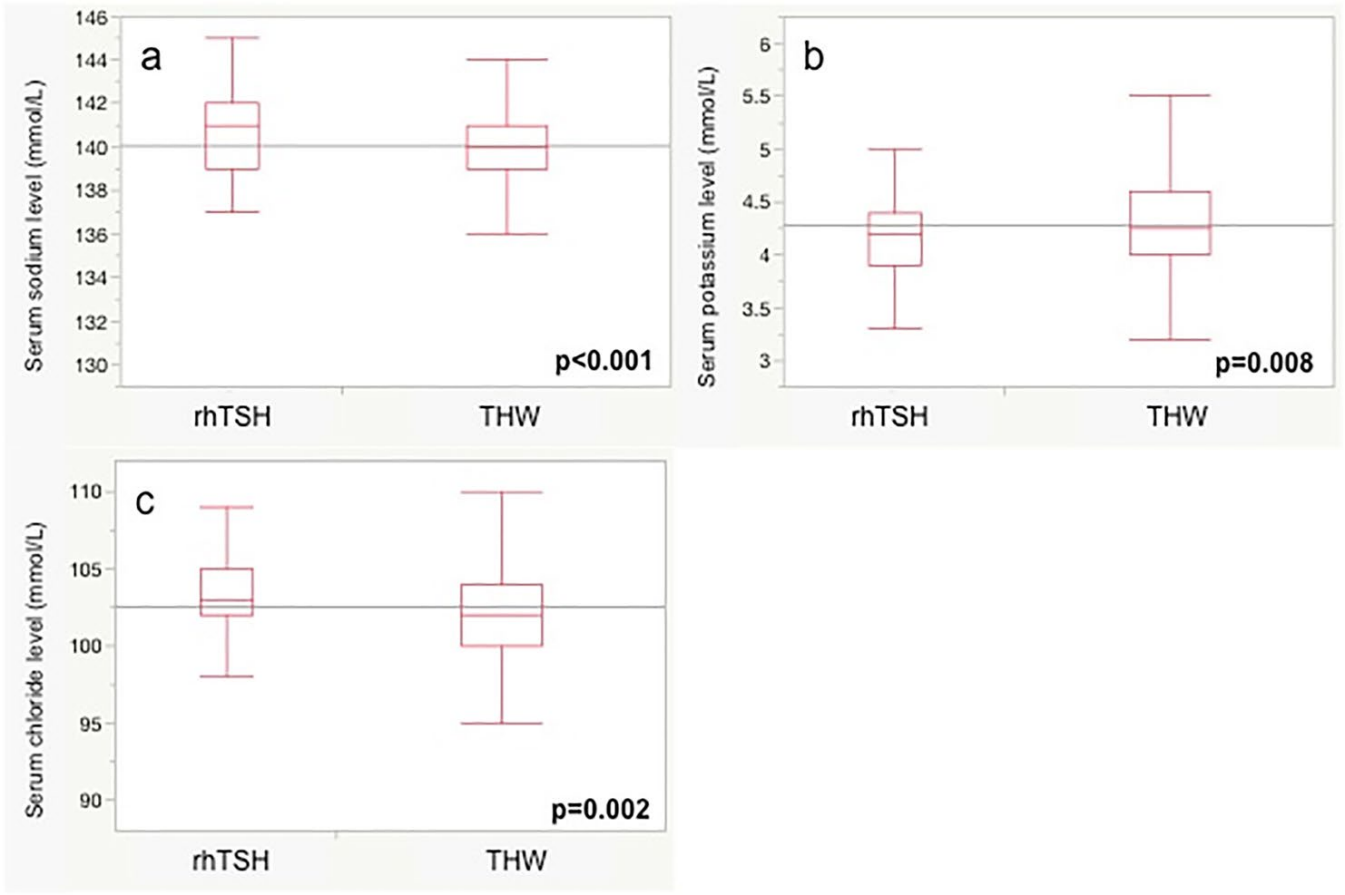
Differences in serum electrolyte levels between the two preparation methods for radioactive iodine therapy. **a** Serum sodium level, **b** serum potassium level, and **c** serum chloride level. *rhTSH* recombinant human thyrotropin, *THW* thyroid hormone withdrawal

Figure 1 shows the differences in thyroid hormones and renal function between the two groups. TSH in the rhTSH group was significantly higher than that in the THW group (p < 0.001). On the other hand, fT4 and fT3 levels in the THW group were significantly lower than those in the rhTSH group (both p < 0.001). Regarding electrolytes (Fig. 2), serum sodium and serum chloride levels in the THW group were significantly lower than those in the rhTSH group (p < 0.001 and p = 0.002, respectively). In contrast, the serum potassium level in the THW group was significantly higher than that in the rhTSH group (p = 0.008).

We also analyzed clinical factors influencing the onset of hyponatremia and hyperkalemia. Table 2 shows the results of the univariate and multivariate analyses of clinical factors influencing the onset of hyponatremia. Age and eGFR were significantly associated with hyponatremia in the univariate analysis (p = 0.033 and p = 0.006, respectively). In the multivariate analysis, only age was significantly associated with hyponatremia (p = 0.030). Table 3 shows the results of the univariate and multivariate analyses of clinical factors influencing the onset of hyperkalemia. Distant metastases, preparation method for RIT and eGFR were significantly associated with the serum potassium level in both the univariate analysis (p = 0.005, p = 0.005 and p = 0.001, respectively). In the multivariate analysis, only eGFR was significantly associated with hyperkalemia (p = 0.019). Table 4 shows the results of the univariate and multivariate analyses of clinical factors influencing renal function impairment (eGFR < 45 mL/min/1.73m^2^) as a reference. Age, distant metastases, using angiotensin-converting enzyme inhibitors (ACEIs) or angiotensin II receptor blockers (ARBs), preparation method for RIT were significantly associated with eGFR in univariate analysis (p = 0.001, p = 0.005, p = 0.033 and p = 0.010, respectively) and only age was significantly associated with eGFR in multivariate analysis (p = 0.013). Tables 5 and 6 show cases of overt hyperkalemia and hyponatremia.

**Table 2:** Univariate and multivariate analyses of clinical factors influencing the onset of hyponatremia

Variable	Univariate			Multivariate		
	P-value	Odds ratio	95% CI	P-value	Odds ratio	95% CI
Age (≥ 65 years)	**0.033**	**	**	**0.030**	5.178 × 10^6^	1.295-infinity
Sex (Female)	0.301	0.398	0.065–2.420			
BMI (≥ 25)	0.143	3.556	0.583–21.68			
Distant metastases (yes)	0.412	2.092	0.344–12.72			
Using ACEIs or ARBs (yes)	0.392	2.167	0.354–13.25			
Prepalation method (THW/rhTSH)	0.989	1.012	0.166–6.157			
eGFR (< 45 mL/min/1.73m^2^)	**0.006**	8.488	1.376–52.37	0.065	5.600	0.891–43.98

*CI* confidence interval, *BMI* body mass index, *ACEIs* angiotensin-converting enzyme inhibitors, *ARBs* angiotensin II receptor blockers, *THW* thyroid hormone withdrawal, *rhTSH* recombinant human thyrotropin, *eGFR* estimated glomerular filtration rate

**Odds ratio of infinity

A p-value <0.05 was considered statistically significant, which is shown in bold

**Table 3:** Univariate and multivariate analyses of clinical factors influencing the onset of hyperkalemia

Variable	Univariate			Multivariate		
	P-value	Odds ratio	95% CI	P-value	Odds ratio	95% CI
Age (≥ 65 years)	0.216	1.747	0.716–4.265			
Sex (female)	0.758	1.151	0.471–2.816			
BMI (≥ 25)	0.619	1.256	0.511–3.087			
Distant metastases (Yes)	**0.005**	3.485	1.385–8.771	0.422	1.591	0.533–5.871
Using ACEIs or ARBs (Yes)	0.806	0.879	0.313–2.466			
Prepalation method (THW/rhTSH)	**0.005**	4.977	1.442–17.17	0.164	2.998	0.629–15.87
eGFR (< 45 mL/min/1.73m^2^)	**0.001**	4.041	1.626–10.04	**0.019**	3.191	1.217–8.035

*CI* confidence interval, *BMI* body mass index, *ACEIs* angiotensin-converting enzyme inhibitors, *ARBs* angiotensin II receptor blockers, *THW* thyroid hormone withdrawal, *rhTSH* recombinant human thyrotropin, *eGFR* estimated glomerular filtration rate

A p-value <0.05 was considered statistically significant, which is shown in bold

**Table 4:** Univariate and multivariate analyses of clinical factors influencing renal function impairment (eGFR < 45 mL/min/1.73m^2^)

Variable	Univariate			Multivariate		
	P-value	Odds ratio	95% CI	P-value	Odds ratio	95% CI
Age (≥ 65 years)	**0.001**	3.158	1.523–6.546	**0.013**	2.529	1.213–5.605
Sex (Female)	0.897	0.957	0.494–1.856			
BMI (≥ 25)	0.185	1.569	0.803–3.066			
Distant metastases (Yes)	**0.005**	2.541	1.311–4.924	0.505	1.371	0.553–3.748
Using ACEIs or ARBs (Yes)	**0.033**	2.091	1.050–4.167	0.112	1.807	0.868–3.678
Prepalation method (THW/rhTSH)	**0.010**	2.627	1.240–5.567	0.242	1.896	0.637–5.413

*eGFR* estimated glomerular filtration rate, *CI* confidence interval, *BMI* body mass index, *ACEIs* angiotensin-converting enzyme inhibitors, *ARBs* angiotensin II receptor blockers, *THW* thyroid hormone withdrawal, *rhTSH* recombinant human thyrotropin

A p-value <0.05 was considered statistically significant, which is shown in bold

**Table 5:** Cases of hyperkalemia (serum potassium level ≥ 5.5 mmol/L)

Case	Age	Sex	Preparation mehod	Distant metastases	eGFR (mL/min/1.73m^2^)	Using ACEIs or ARBs	Serum potassium level (mmoL/L)
1	60	Male	THW	Lung	51.4	No	6.1
2	79	Female	THW	Lung	54.5	No	5.6
3	72	Male	THW	Lung	40.0	Yes	5.7
4	78	Female	THW	Lung	60.7	No	5.5

*eGFR* estimated glomerular filtration rate, *ACEIs* angiotensin-converting enzyme inhibitors, *ARBs* angiotensin II receptor blockers, *THW* thyroid hormone withdrawal

**Table 6:** Cases of hyponatremia (serum sodium level < 134 mmol/L)

Case	Age	Sex	Preparation mehod	Distant metastases	eGFR (mL/min/1.73m^2^)	Using ACEIs or ARBs	Serum sodium level (mmoL/L)
1	76	Male	THW	LN	40.0	Yes	132
2	82	Male	rhTSH	No	35.9	No	133
3	72	Female	rhTSH	No	25.0	Yes	130
4	70	Male	THW	LN, lung	54.9	No	133
5	69	Female	THW	Lung	57.2	No	133

*eGFR* estimated glomerular filtration rate, *ACEIs* angiotensin-converting enzyme inhibitors, *ARBs* angiotensin II receptor blockers, *THW* thyroid hormone withdrawal, *rhTSH* recombinant human thyrotropin, *LN* lymph node

## Discussion

Previous reports have suggested that there are advantages to using the rhTSH method. For example, the rhTSH method results in fewer symptoms associated with hypothyroidism than THW [10]. Hypothyroidism causes renal function impairment [2], and a decreased GFR during THW results in higher radiation exposure [13]. Moreover, serum electrolyte disorder caused by hypothyroidism has also been reported [3, 4]. However, a comparison of the serum electrolyte changes caused by the two preparation methods for RIT has not been published. Therefore, we believe that our study may provide new information about electrolyte changes with THW and rhTSH.

Our study showed that serum potassium was significantly higher in the THW group than in the rhTSH group. Regarding the clinical factors of overt hyperkalemia (≥ 5.0 mmol/L), distant metastases, eGFR and preparation method were significantly associated with hyperkalemia in univariate analysis, however, only eGFR was significantly different in multivariate analysis. The kidney is an important organ for maintaining potassium levels, and the regulation of potassium excretion in the distal nephron is affected by circulating aldosterone [14]. Aldosterone activates sodium and potassium transport along the aldosterone-sensitive distal nephron [14]. When the loss of kidney mass occurs, Na–K-ATPase activity is stimulated [14]. However, exogenous load activities are limited when hyperkalemia develops because of decreased distal sodium delivery [14]. Accordingly, renal function impairment is one of the causes of hyperkalemia. In our study, eGFR was significantly lower in the THW group than in the rhTSH group; meanwhile, the serum potassium level was significantly higher in the THW group than in the rhTSH group. Considering that thyroid hormone affects renal function [2], the THW method may affect serum potassium elevation by influencing renal function. In our institution, four patients had a serum potassium level ≥ 5.5 mmol/L; their ECG findings did not reveal any abnormalities like T waves and widened QRS complexes which are observed in severe hyperkalemia, and no treatment for hyperkalemia was administered. Subsequently, their serum potassium level decreased spontaneously, and hyperkalemia was not observed during follow-up. These patients did not have severe renal failure, and all of them underwent RIT following THW. Based on our findings, we may need to pay more attention to serum potassium level, as well as renal function, during THW for RIT.

Serum sodium level was significantly lower in the THW group than in the rhTSH group. Regarding the clinical factors of overt hyponatremia (< 134 mmol/L), renal function and older age were significantly associated with hyponatremia, whereas preparation method was not. In our study, overt hyponatremia was observed in both groups; these five patients were all aged 65 years and older. Previous studies reported cases of older patients with severe hyponatremia during RIT [15, 16]. Besides, other reports showed that older age was related to hyponatremia [3, 17]. Our findings are consistent with the findings of these studies, i.e., older patients may have a higher risk of hyponatremia. Thus, older patients might be more sensitive to high vasopressin which can cause hyponatremia than younger patients [18], and serum sodium levels should be carefully monitored in older patients during RIT.

Drinking a lot of water for renal protection may lead to hyponatremia, and cases of severe and life-threatening hyponatremia within 1 week after RIT have been reported [3]. We did not take blood samples after treatment to avoid radiation exposure to medical workers; therefore, serum sodium level after RIT was not analyzed. Because patients start to drink water on the day of RIT, more severe hyponatremia might only occur after RIT. In the present study, no patient had hyponatremia during follow-up; however, the likelihood of severe hyponatremia after RIT needs to be recognized, especially in patients at risk of hyponatremia such as older patients. If hyponatremia is observed before RIT, attention should be paid not only to water intake but also to sodium intake.

In addition, other reasons for hyponatremia have been suggested. To perform RIT properly, not only elevations in TSH but also a reduction in iodine through a low-iodine diet is essential. Iodized salt is used in many countries to prevent iodine deficiency [19, 20]. Therefore, a low-iodine diet can lead to sodium restriction in these countries, which may become a trigger for hyponatremia [21]. On the other hand, iodized salt is not generally used in Japan because iodine deficiency is hardly ever observed in Japanese people eating a seafood-rich diet. Hence, we cannot apply the type of low-iodine diet used in other countries to patients in Japan. Moreover, whether a low-iodine diet in Japan changes sodium intake is unknown. It has been reported that a low-iodine diet during RIT is not a risk factor for severe hyponatremia [22]; thus, not all patients are at risk of hypernatremia with a low-iodine diet. However, sodium intake during RIT might need to be assessed if patients have other risk factors for hyponatremia. Recently, it was suggested that a shorter duration of a low-iodine diet may be sufficient for RIT [23]; thus, the period of the low-iodine diet in high-risk patients might need to be re-evaluated.

Our study showed that serum chloride level was significantly lower in the THW group than in the rhTSH group. Chloride is the most important anion to maintain serum osmolarity and fluid balance, which interacts with serum sodium [24]. This may be the reason why the change in serum chloride was similar to the one in serum sodium. However, there were no reports which discussed change in serum chloride during RIT. Hypochloremia has been reported as a prognostic factor in patients with heart failure, meanwhile the role of chloride as a therapeutic target remains unclear [25]. Besides, as opposed to hyponatremia and hyperkalemia, hypochloremia itself is not a fatal condition. Therefore, this electrolyte abnormality may have been overlooked. Nevertheless, serum chloride level as well as serum sodium level may need to be monitored during RIT.

Previous studies suggested that ACEIs and ARBs were risk factors for hyponatremia and hyperkalemia [26, 27]. These drugs inhibit the renin–angiotensin–aldosterone system which blocks aldosterone secretion from the adrenal glands [14]. Therefore, sodium absorption and potassium excretion from the distal nephron are affected which can lead to hyponatremia and hyperkalemia [14, 26, 27]. In our study, using ACEIs or ARBs was not significantly associated with overt hyperkalemia and hyponatremia. Although the effect of these drugs appears to be limited, they may promote electrolyte abnormalities if patients have other risk factors.

This study has several limitations. First, this was a retrospective study, and the dates when we obtained blood samples were not consistent. To get more precise results, blood samples should be obtained on the day of RIT. Second, blood samples before RIT were not used in our study because the analysis was retrospective; therefore, we could not compare the data of patients at different times. Consequently, renal function before RIT was not considered, and the analyses might be inadequate to describe changes in renal function and serum electrolytes. Thus, further prospective studies including these data are needed to solve this problem. Third, eGFR may not precisely reflect renal function under the condition of hypothyroidism because eGFR was calculated using serum creatinine. Since serum creatinine is affected by muscle volume, patients with higher muscle mass have relatively lower eGFR [28]. Therefore, eGFR in our study may not be reflective of renal function. Besides, hypothyroidism can cause muscle dysfunction such as pain, cramping, and slow reflexes [29], and it has been reported that muscle mass is reduced during hypothyroidism [30]. Moreover, the effect of temporary hypothyroidism on serum creatinine is unclear. Nevertheless, we need to take this effect into consideration when describing eGFR in patients undergoing THW. To remove the effect of muscle mass, eGFR can be calculated using serum cystatin C [31]. However, serum cystatin C is lower under the condition of hypothyroidism in contrast with serum creatinine [31]. Therefore, eGFR calculated using cystatin C may appear good. If renal function status needs to be determined precisely, inulin clearance might be the best method, as it is the reference method for GFR measurement [32]. However, this method is complicated and requires a long time making it challenging to implement in clinical practice. Therefore, we think that it is reasonable to use serum creatinine for calculating the eGFR. Finally, this retrospective study didn’t examine treatment effects of RIT due to lack of patient information during follow-up periods. To evaluate effects of preparation methods more accurately, we need to discuss treatment effects of RIT as well as side effects. It may also need to be evaluated in further studies.

## Conclusion

THW and rhTSH for RIT affect serum sodium and potassium levels differently. THW may be associated with overt hyperkalemia. Renal function may be risk factors for hyperkalemia, whereas older age may be a risk factor for hyponatremia.
